# Effect of *sp*^*3*^*/sp*^*2*^ carbon ratio and hydrodynamic size on the biodistribution kinetics of nanodiamonds in mice *via* intravenous injection

**DOI:** 10.1186/s12989-023-00545-7

**Published:** 2023-08-21

**Authors:** Jiyoung Jeong, Soyeon Jeon, Songyeon Kim, Sinuk Lee, Gyuri Kim, Eunsol Bae, Yeonjeong Ha, Seung Whan Lee, Ji-Su Kim, Dong-Jae Kim, Wan-Seob Cho

**Affiliations:** 1 Lab of Toxicology, Department of Health Sciences, Dong-A University, Busan, 49315 Republic of Korea; 2 Plasma Technology Research Center, National Fusion Research Institute, Gunsan-si, 54004 Republic of Korea; 3 Primate Resources Center (PRC), Korea Research Institute of Bioscience and Biotechnology (KRIBB), Jeongeup, 56216 Republic of Korea; 4 Laboratory Animal Resource Center, DGIST, Daegu, 42988 Republic of Korea

**Keywords:** Nanodiamond, Toxicokinetic, *sp*^*3*^*/sp*^*2*^ carbon ratio, Hydrodynamic size, Accumulation

## Abstract

**Background:**

Nanodiamonds (NDs) have gained a rapidly growing interest in biomedical applications; however, little is known regarding their biokinetics owing to difficulties in measurements and limited synthesis/purification technologies. In this study, we investigated the distribution kinetics of detonation-synthesized NDs in mice *via* intravenous injection to evaluate the parameters that determine the behavior of the particles. We prepared two distinctive NDs that controlled the *sp*^*3*^*/sp*^*2*^ carbon ratio and particle size by coating them with serum proteins. The four control samples were intravenously injected into mice, and tissue distribution and clearance were evaluated at 30 min and 1, 7, and 28 days post-injection.

**Results:**

The *sp*^*3*^*/sp*^*2*^ carbon ratio showed no correlation with the organ distribution of the NDs. However, hydrodynamic size showed an excellent correlation with organ distribution levels: a negative correlation in the liver and positive correlations in the spleen and lungs. Furthermore, the deposition levels of NDs in the lung suggest that particles smaller than 300 nm could avoid lung deposition. Finally, a similar organ distribution pattern was observed in mice injected with carbon black nanoparticles controlled hydrodynamic size.

**Conclusions:**

In conclusion, the tissue distribution of NDs is modulated not by the *sp*^*3*^*/sp*^*2*^ carbon ratio but by the hydrodynamic size, which can provide helpful information for targeting the tissue of NDs. Furthermore, the organ distribution pattern of the NDs may not be specific to NDs but also can apply to other nanoparticles, such as carbon black.

**Supplementary Information:**

The online version contains supplementary material available at 10.1186/s12989-023-00545-7.

## Introduction

The rapid advancement of nanomedicine has facilitated the development of various nanomaterials for various applications, including agents for delivering, sensing, imaging, and therapeutics. Among nanomaterials, carbon nanomaterials are attractive for various applications owing to their biocompatibility and unique physicochemical properties, such as superior strength, flexibility, and high electrical conductivity [1, 2]. Nanodiamonds (NDs) are among the most favorable materials for biomedical applications owing to their excellent biocompatibility and versatility [3–6]. For example, recent studies have reported that NDs can target a specific cancer cell organelle, circumvent drug resistance by conjugating ligands [7], or resolve inflammation by conjugating an anti-inflammatory drug [8].

Surface modification in nanomedicine can offer multiple benefits in various ways; however, it can also alter physicochemical properties such as size, shape, surface chemistry, and agglomeration status, which can cause undesirable effects [9–11]. Several studies have demonstrated that these physicochemical properties can influence blood circulation time, protein corona formation, and cellular internalization, leading to its unique biological behavior, including toxicity and biodistribution [12–16]. For example, nanoparticles conjugated with the longer chain of polyethylene glycol (PEG) showed a prolonged blood circulation time and reduced uptake to reticuloendothelial system (RES) organs, which highlights the role of PEG chain length on biodistribution [17]. In addition, the intravenous injection of gold nanoparticles of different sizes showed that smaller (e.g., 10 nm) particles have a wider tissue distribution and larger (e.g., 50, 100, and 250 nm) particles have a higher deposition in the liver and spleen [18].

The pulmonary administration of NDs to rats in our previous study showed that all tested NDs had a low inflammatory potential and decreased inflammation by increasing the *sp*^*3*^ carbon ratio [19]. Thus, the biological effect of the *sp*^*3*^*/sp*^*2*^ carbon ratio can provide information for developing safer NDs for biomedical applications [14, 20, 21]. As an expansion of our previous study, we investigated the role of the *sp*^*3*^*/sp*^*2*^ carbon ratio and hydrodynamic size on the tissue distribution and clearance kinetics in mice after intravenous injection using two types of NDs (i.e., ^low^ND for ND with low *sp*^*3*^*/sp*^*2*^ carbon ratio; ^high^ND for ND with high *sp*^*3*^*/sp*^*2*^ carbon ratio) with different dispersion modifications (i.e., poorly dispersed and well-dispersed).

## Results

### Physicochemical characterization of NDs

**Fig. 1:**
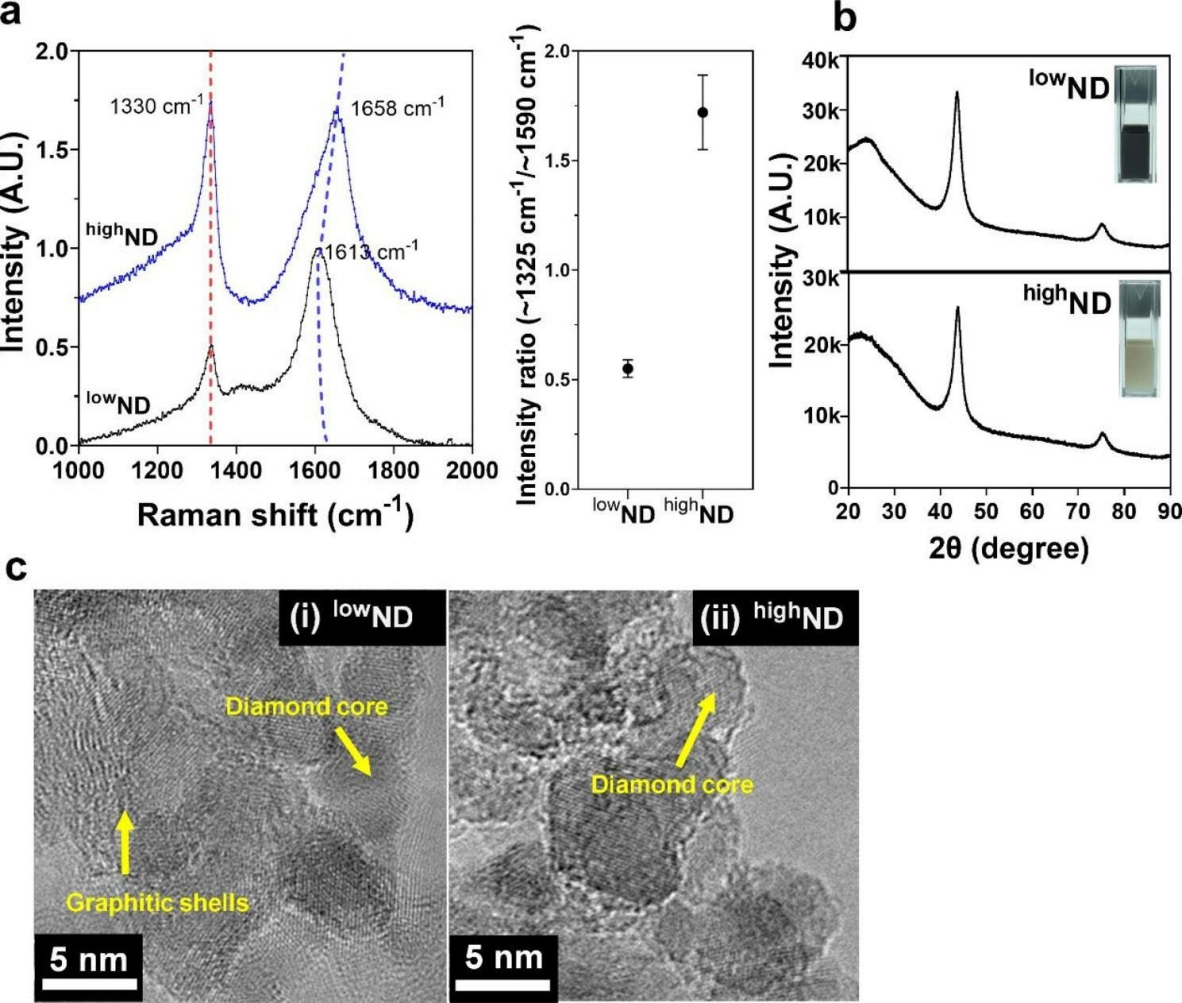
UV-Raman spectra, XRD, and HR-TEM images of low *sp*^*3*^*/sp*^*2*^ NDs (^low^ND) and high *sp*^*3*^*/sp*^*2*^ NDs (^high^ND). (**a**) UV-Raman spectra and intensity ratio (*I*_Dia_/*I*_G_ = 1325 cm^− 1^/1590 cm^− 1^). (**b**) The XRD patterns show clear diamond peaks and graphite peaks. (**c**) HR-TEM images show that ^low^ND has thicker graphitic shells than ^high^ND. The photomicrographs inserted in Fig. 1b show a color change from black-colored ^low^ND to gray-colored ^high^ND

The primary particle size, surface area, *sp*^*3*^*/sp*^*2*^ carbon ratio, hydrodynamic size, polydispersity, and surface charge are presented in Table 1. The Raman spectra and X-ray diffraction (XRD) and high-resolution transmission electron microscopy (HR-TEM) images are shown in Fig. 1. As ^low^ND and ^high^ND originated from the same, the surface area and primary size were similar (surface area: 270–275 m^2^/g; primary size: 4.86–4.93 nm). The *sp*^*3*^*/sp*^*2*^ carbon ratio of the NDs was expressed as the intensity ratio (*I*_Dia_/*I*_G_) of the ultraviolet Raman spectra between the 1325 cm^− 1^ diamond peak (*I*_Dia_) and 1590 cm^− 1^ G band peak (*I*_G_). The *I*_Dia_/*I*_G_ ratio of ^low^ND and ^high^ND were 0.55 ± 0.04 and 1.72 ± 0.17, respectively. In addition, the XRD pattern exhibited three peaks at 2*θ* = 43.8° (111), 75.2° (220), and 91.1° (311), which were commonly observed in the ^low^ND and ^high^ND samples, whereas a broad peak near 2*θ* = 26° was observed in the ^low^ND samples (Fig. 1b). The HR-TEM image showed that ^low^ND has a higher number of graphitic shells covering the diamond cores than the ^high^ND (Fig. 1c). The hydrodynamic sizes of ^low^ND and ^high^ND suspended in phosphate-buffered saline (PBS) showed that both particles were agglomerated (515.8–629.7 nm) because of the hydrophobic nature of the particles. No significant differences in the hydrodynamic size between ^low^ND and ^high^ND were observed. However, the dispersion of particle samples using 3% mouse serum significantly improved particle dispersion. The hydrodynamic sizes of serum-coated ^low^ND (^low^SND) and serum-coated ^high^ND (^high^SND) were 303.4 ± 2.7 and 269.0 ± 1.7 nm, respectively. Although the zeta potentials of NDs in PBS were different from each other (-5.23 mV for ^low^ND and − 23.90 mV for ^high^ND), those of serum-coated particles were similar (-11.60 mV for ^low^SND and − 13.20 mV for ^high^SND) (Table 1).

**Table 1:** Physicochemical properties of NDs used for animal experiments

Physicochemical parameters	Types of ND	
	^low^ND	^high^ND
Primary size (nm)	4.93 ± 0.4	4.86 ± 0.3
Surface area (m^2^/g)	270.2	275.4
*sp*^*3*^*/sp*^*2*^ carbon ratio (*I*_Dia_/*I*_G_)	0.55 ± 0.04	1.72 ± 0.17
Hydrodynamic size (nm) in		
PBS	629.7 ± 34.0	515.8 ± 28.4
PBS with 3% mouse serum	303.4 ± 2.7	269.0 ± 1.7
Polydispersity index in		
PBS	0.33 ± 0.04	0.23 ± 0.02
PBS with 3% mouse serum	0.32 ± 0.01	0.14 ± 0.03
Zeta potential (mV) in		
PBS	-5.23 ± 0.22	-23.90 ± 0.53
PBS with 3% mouse serum	-11.60 ± 0.17	-13.20 ± 0.44

The data were expressed as mean ± SEM

### The surface charge and hydrodynamic size of ND samples before and after incubation with mouse plasma

**Fig. 2:**
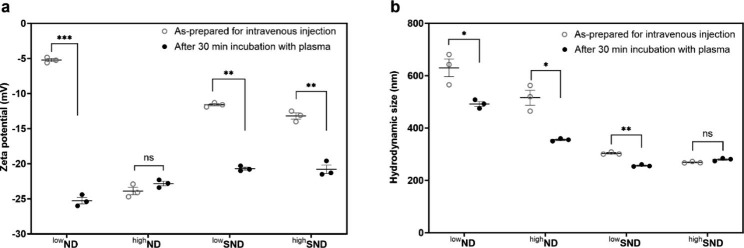
The surface charge and hydrodynamic size of ND samples before and after incubation with mouse plasma. The as-prepared particles for intravenous injection were incubated for 30 min with mouse plasma, and zeta potential and hydrodynamic size were measured after redispersing them in DW. (**a**) The diverse zeta potentials of all as-prepared ND samples for intravenous injection were within a similar range from − 25 mV to -20 mV. (**b**) Although the hydrodynamic sizes of all as-prepared ND samples for intravenous injection were reduced after incubation with plasma, the pattern of size distribution was consistent. Data are expressed as mean ± SEM for each group (*n* = 3 per group)

In this study, we hypothesized that both NDs with/without serum coating would end up with protein adsorption onto their surface when injected intravenously, and the only difference is the agglomeration size (Fig. S1, see Supporting Information). This hypothesis was proved by measuring the changes in zeta potential and hydrodynamic size of ND samples before and after incubation with mouse plasma to show how the as-prepared particles changed their size and zeta potential after interacting with mouse blood. The result showed that the diverse zeta potentials around − 25 mV to -5 mV of all ND samples became similar around − 25 mV to -20 mV after incubation with plasma (Fig. 2a). Interestingly, the zeta potentials around − 13 mV to -11 mV of ^low^SND and ^high^SND were further decreased to around − 21 mV, possibly due to the further protein adsorption on the serum protein-coated ND (Fig. 2a). The hydrodynamic sizes of as-prepared ^low^ND and ^high^ND for intravenous injection were significantly reduced after incubation with plasma (Fig. 2b). It should be noted that the hydrodynamic size measurement of incubation with plasma can provide limited information because particles were sonicated when redispersing them in distilled water (DW) after incubation with plasma. Nevertheless, the hydrodynamic sizes of 4 particle samples showed that the as-prepared particles for intravenous injection might keep their hydrodynamic size, as hypothesized in this study (Fig. S1, see Supporting Information).

### Organ distribution kinetics of NDs in mice

**Fig. 3:**
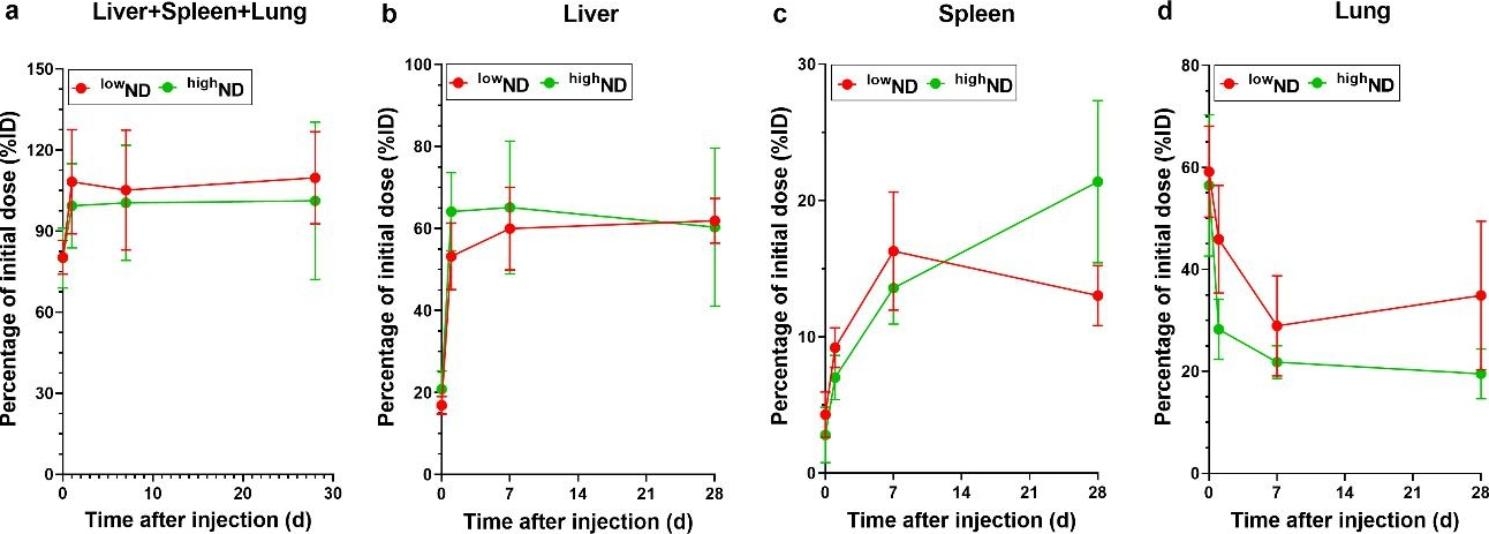
Time-course organ distribution pattern of NDs in the major accumulating organs. (**a**) The combined ND levels in the liver, spleen, and lung show constant levels from day 1 to day 28. (**b**) The ND levels in the liver show saturation levels of about 50–70% of the initial dose (ID) from day 1 to day 28. (**c**) The ND levels in the spleen show an increasing pattern up to day 28. (**d**) The ND levels in the lung show a decreasing pattern from 30 min post-injection (57% ID) to day 28 (27% ID). Data are expressed as mean ± SEM (*n* = 5) for each group

The suspensions of ^low^ND and ^high^ND were injected into mice *via* the tail vein to compare the organ distribution kinetics of two different *sp*^*3*^*/sp*^*2*^ ratio NDs dispersed in PBS (hydrodynamic size: 515.8–629.7 nm). The concentrations of NDs in the organs were quantified at 30 min and 1, 7, and 28 days post-treatment. Quantification of NDs was successful by proteinase K (PK) tissue digestion and UV-Vis spectrophotometry at 750 nm (Fig. S2, see Supporting Information). The organs accumulating NDs were the liver, spleen, and lungs throughout the tested time points. The accumulating property of NDs was highlighted by the fact that the combined levels of NDs at 30 min post-injection in these three organs were approximately 80.20% of the initial injection dose, reached approximately 103.82% from day 1, and persisted up to day 28 (Fig. 3a). Furthermore, the total amount of NDs in these three organs was not significantly different based on the *sp*^*3*^*/sp*^*2*^ carbon ratio (Fig. 3a). The NDs in the liver at 30 min showed 18.84% of the initial dose (ID) and reached a saturated phase (58.63% of the ID) from day 1 (Fig. 3b). However, the levels of NDs in the spleen showed a gradual accumulation pattern from 30 min (4.29% and 2.81% of the ID for ^low^ND and ^high^ND, respectively) to day 28 (13.02% and 21.38% of the ID for ^low^ND and ^high^ND, respectively) (Fig. 3c). In contrast, the levels of NDs in the lungs showed a decreasing tendency from 30 min post-injection (59.15% and 56.47% of the ID for ^low^ND and ^high^ND, respectively) to day 28 post-injection (34.89% and 19.51% of the ID for ^low^ND and ^high^ND, respectively) (Fig. 3d). In addition, the reduction percentages of ND levels in the lung on day 28 compared to 30 min post-injection were 24.26 and 36.96% for ^low^ND and ^high^ND, respectively (Fig. 3d). However, the concentration of NDs in other organs, including the kidney, mesenteric lymph nodes, heart, and brain, was below the limit of quantification (LOQ:10 µg/mL for both particles) (Table S1, see Supporting Information).

### Toxicokinetic parameters of NDs

The toxicokinetic parameters of NDs in the liver, spleen, and lungs are presented in Table 2. The calculated toxicokinetic parameters, including concentration max (C_max_) and area under the curve (AUC) of NDs in these three organs, were not significantly different based on the *sp*^*3*^*/sp*^*2*^ carbon ratio, but differed by organ type. The C_max_ was in the order of liver, lung, and spleen. Although the numeric values of time max (T_max_) in the liver were significantly different based on the *sp*^*3*^*/sp*^*2*^ carbon ratio, the graphical evaluation of T_max_ in the liver was not significantly different between the two ND types owing to the constant levels. The T_max_ in the liver and spleen from the visual assessment was approximately 1 and 28 days, respectively (Fig. 3b, c). However, the T_max_ in the lung was 30 min post-injection. As NDs showed no significant clearance pattern from the accumulating organs, the AUC data showed an increasing linear pattern (Table 2, Fig. S3, see Supporting Information). Meanwhile, the values of AUC per organ were in the order of liver, lung, and spleen, whereas AUC per organ weight was in the order of lung, spleen, and liver (Fig. S3, see Supporting Information).

**Table 2:** The toxicokinetic parameters in the liver, spleen, and lung

Parameters	C_max_ (µg)	T_max_ (h)	AUC_0 − 1d_	AUC_0 − 7d_	AUC_0 − 28d_
^low^ND					
- Liver	309.3 ± 27.3	672	175.1	1872.0	8269.0
- Spleen	81.4 ± 21.7	168	33.7	416.2	1955.0
- Lung	295.8 ± 44.7	0.5	262.6	1385.0	4735.0
^high^ND					
- Liver	325.5 ± 80.9	168	212.2	2150.0	8735.0
- Spleen	106.9 ± 29.8	672	24.6	333.4	2168.0
- Lung	282.4 ± 69.3	0.5	211.8	962.5	3132.0

Data were expressed as mean ± SEM. The AUC was calculated based on concentration(µg)/organ

### Histopathology in the accumulating organs

Histopathological evaluation was performed for three accumulating organs: the liver, spleen, and lungs. On day 1 post-injection, no treatment-related histological alterations were observed in these organs (Fig. S4, see Supporting Information). The liver, spleen, and lung tissues at day 28 showed normal histological features comparable to those of the vehicle control group (Fig. S4, see Supporting Information).

### Visualization of NDs in organs using the dark-field microscopy

**Fig. 4:**
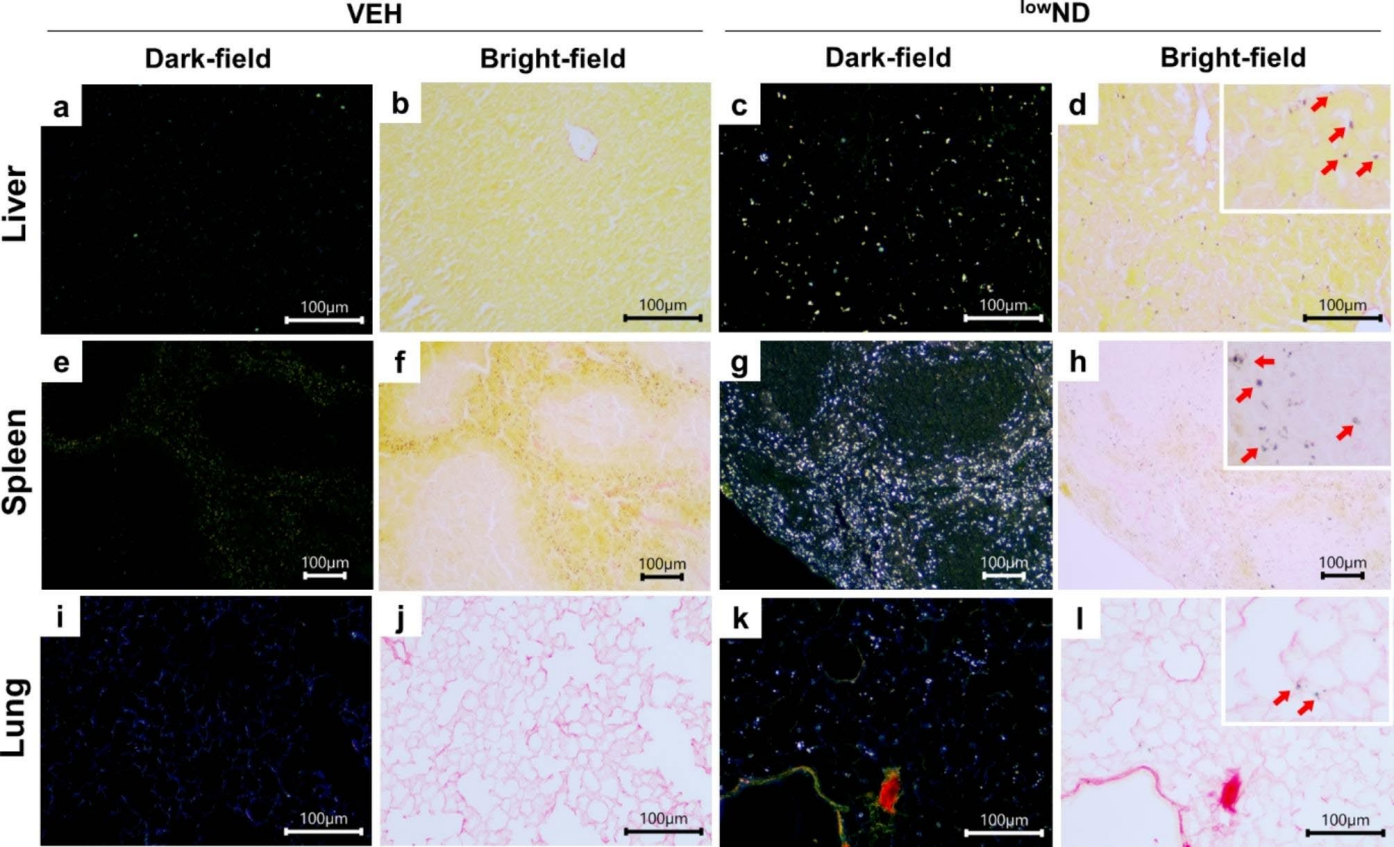
Representative dark-field and bright-field images of the liver, spleen, and lung at day 7 post-injection of ^low^ND. Liver section of mice in the (**a**, **b**) vehicle control (VEH) group and (**c**, **d**) ^low^ND group. Spleen section of mice in the (**e**, **f**) VEH group and (**g**, **h**) ^low^ND group. Lung section of mice in the (**i**, **j**) VEH group and (**k**, **l**) ^low^ND group. The red arrows in the insert image of Fig. 4d, h, and i show that the NDs were located in the liver sinusoids (engulfed by Kupffer cells), red pulps in the spleen, and capillaries in the lungs. The dark-field and bright-field images were taken from the same slides. Scale bar = 100 μm

Dark-field microscopy was used to evaluate organ distribution patterns and tissue localization. The dual observation of NDs with dark-field microscopy and light microscopy in tissue slice sections stained with picrosirius red (PSR) can successfully indicate the histological localization of particles in the liver, spleen, and lung. The particles in the liver were mainly deposited in the sinusoid and engulfed by Kupffer cells (Fig. 4c, d). In contrast, NDs in the spleen were massively distributed to red pulps, located inside macrophage-like cells and the extracellular matrix (Fig. 4 g, h). In addition, the NDs in the lungs were scattered in the region of the alveolar capillaries (Fig. 4k, l; Fig. S5, see Supporting Information for ^high^ND); however, further TEM observation is needed to evaluate the precise location.

### Tissue localization of NDs in the lung by TEM

**Fig. 5:**
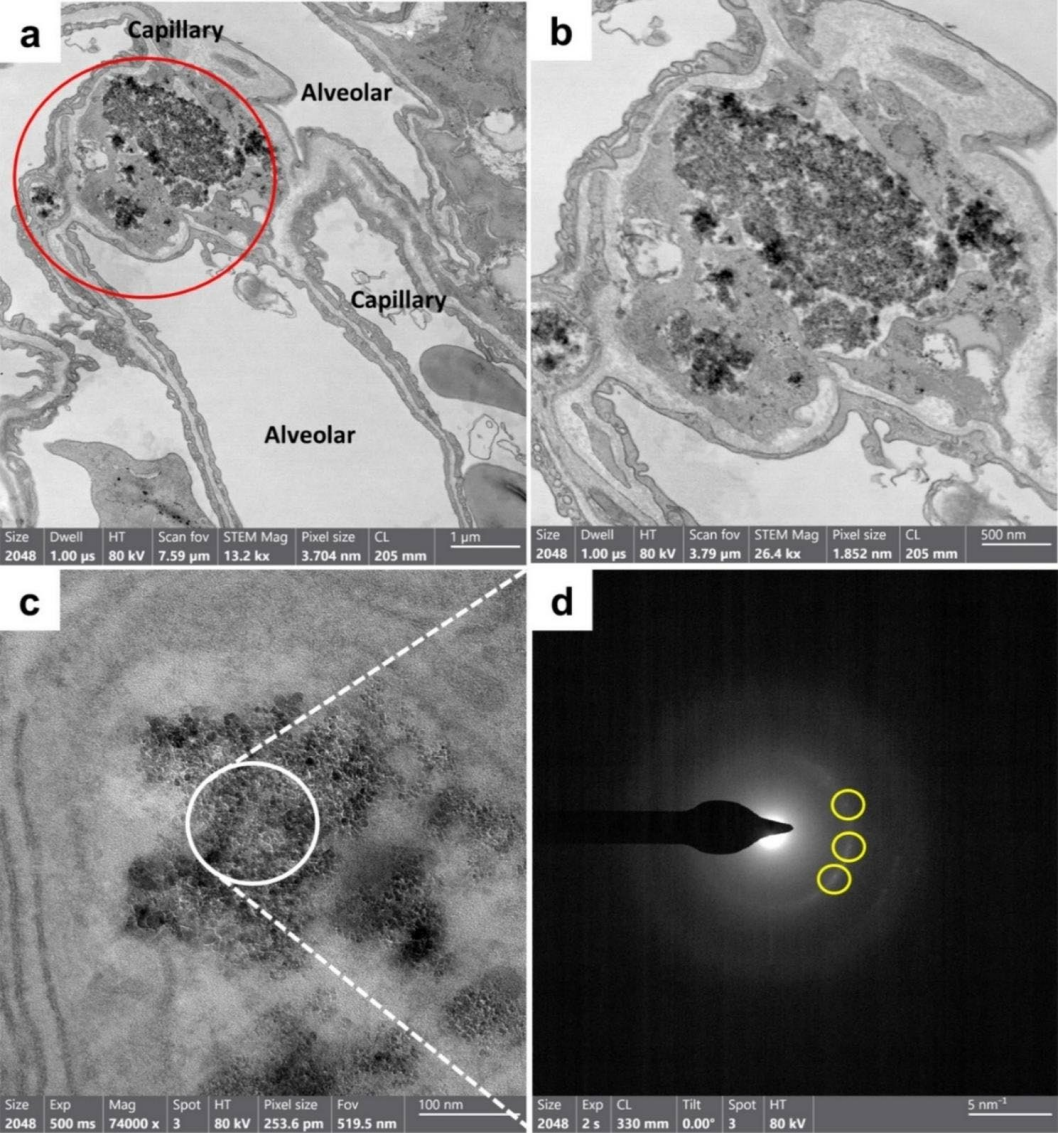
Representative TEM images of the lung at day 7 post-injection of ^low^ND. (**a**) ^low^ND are clustered in alveolar capillaries. (**b**) The higher magnification images of the red circle area of Fig. 4a suggest particles are stuck with the proteinous materials. (**c**) The higher magnification images of Fig. 4b. (**d**) SAED pattern of the ND cluster indicates electron diffraction spots (yellow circles), which suggests that particles are exogenous with the crystal structure

The lung tissue was observed by TEM because of the vague location in the alveolar capillaries observed by dark-field and bright-field microscopy. TEM observations showed that the NDs in the lungs were located in alveolar capillaries (Fig. 5). Interestingly, the NDs were stuck with proteinous materials, which did not detach from the capillary endothelium. The Selected-Area Electron Diffraction (SAED) pattern confirmed that the black particles were exogenous nanoparticles, which must be NDs (Fig. 5d).

### The hydrodynamic size-dependent distribution of NDs in the lung

**Fig. 6:**
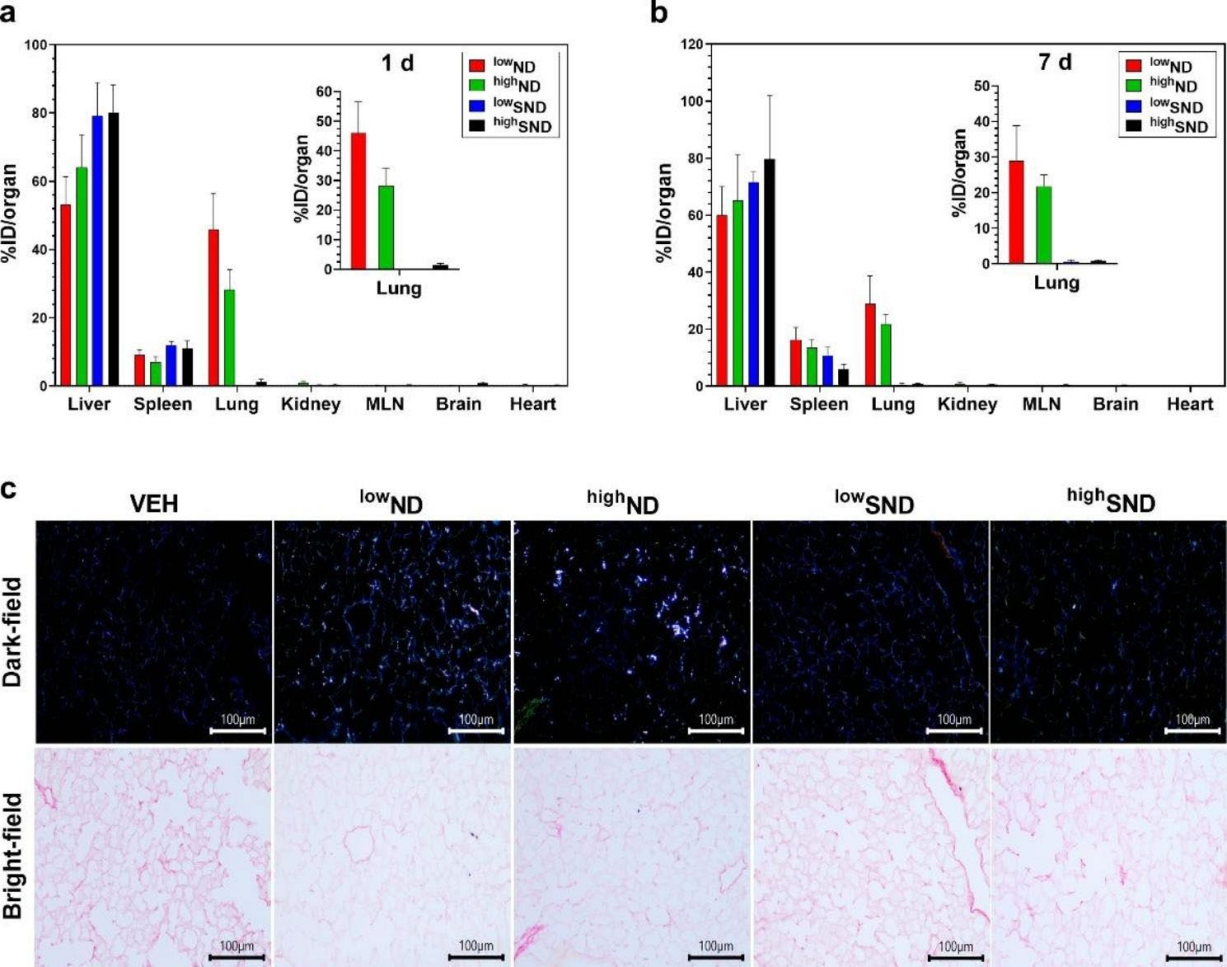
Comparison of the lung distribution of NDs without serum coating (^low^ND and ^high^ND) and with serum coating (^low^SND and ^high^SND). The time-point for comparison was on (**a**) day 1 and (**b**) day 7 post-injection. (**c**) Representative dark-field and bright-field images of NDs in the lung at day 7 post-injection. The data are expressed as mean ± SEM and *n* = 5 for each group. Lung tissue was stained with PSR and scale bar = 100 μm. MLN, mesenteric lymph node

As the *sp*^*3*^*/sp*^*2*^ carbon ratio was not a determinant of the tissue distribution of NDs, we further evaluated the impact of hydrodynamic size by comparing the tissue distribution of NDs after dispersion with serum protein. The hydrodynamic size of ^low^SND and ^high^SND was 303.4 ± 2.7 and 269.0 ± 1.7 nm, respectively (Table 1). Consistent with the organ distribution data of NDs without serum coating, the data of NDs with serum coating showed no significant difference in organ distribution owing to variations in the *sp*^*3*^*/sp*^*2*^ carbon ratio. However, the hydrodynamic size effectively affected the lung distribution of the NDs because the massive deposition of particles without serum coating was significantly reduced in the NDs after coating with serum proteins (Fig. 6).

### The correlation of the hydrodynamic size and organ distribution of NDs

**Fig. 7:**
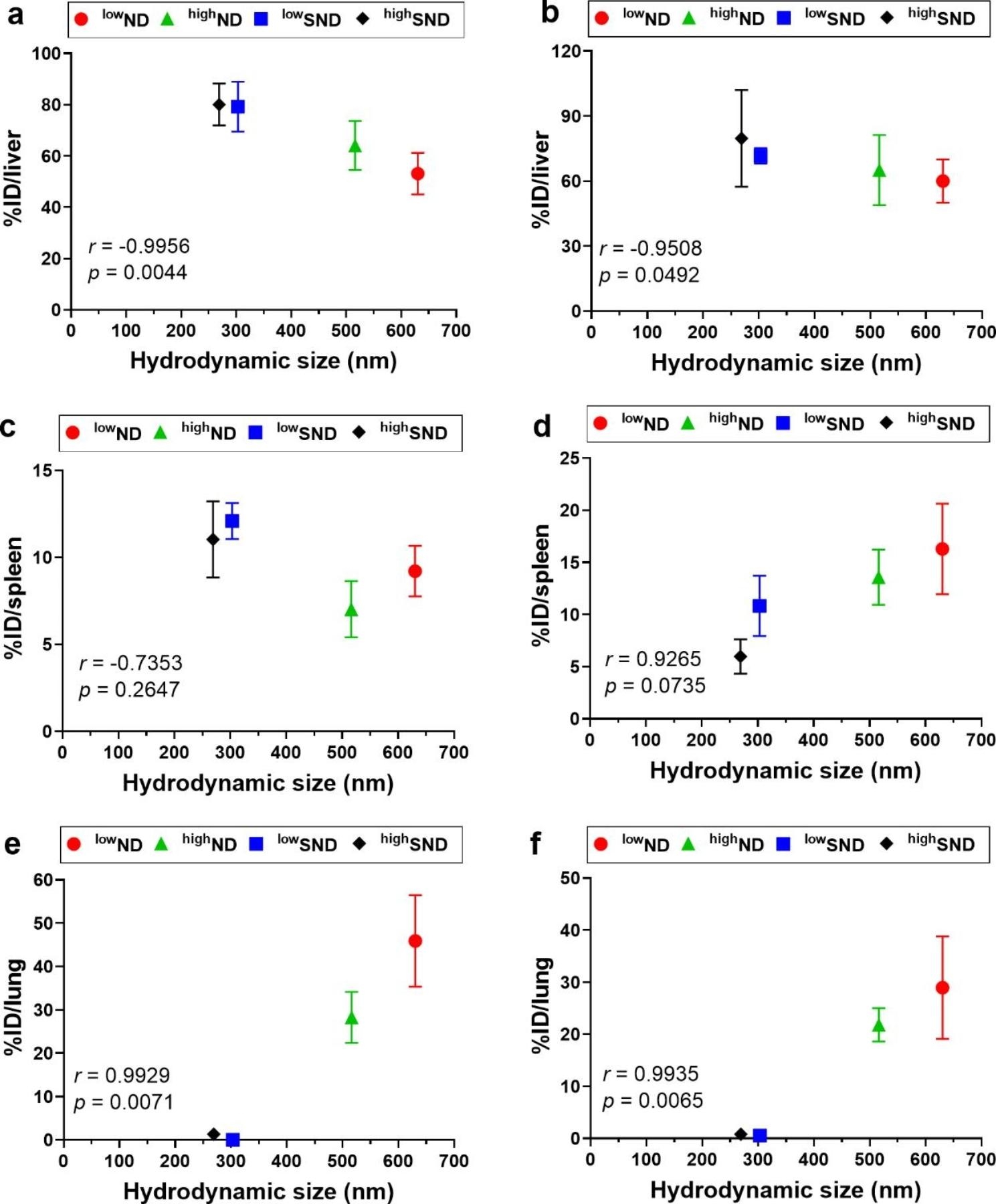
The Pearson correlation test of the hydrodynamic size of NDs against the percentage of initial treatment dose per organ (%ID/organ). A correlation was tested for the liver on (**a**) day 1 and (**b**) day 7, spleen on (**c**) day 1 and (**d**) day 7, and lung on (**e**) day 1 and (**f**) day 7 post-injection. Data are expressed as mean ± SEM for each group (*n* = 5 per group). A two-tailed Pearson correlation test was applied, and the correlation coefficient and *p*-values are indicated in each figure

Pearson’s correlation analysis was performed to evaluate the impact of hydrodynamic size on tissue distribution levels. In the liver, the hydrodynamic size showed a significant negative correlation with the percentage of initial dose (%ID) in organs on days 1 and 7 post-injection (Fig. 7a, b). In the spleen, the hydrodynamic size showed a negative trend with %ID on day 1 post-injection (Fig. 7c) but a positive trend on day 7 post-injection (Fig. 7d), although both time points showed no statistical significance. Lastly, in the lungs, hydrodynamic size showed an apparent positive correlation with %ID at both time points (*r* = 0.9929, *p* = 0.0071 on day 1; *r* = 0.9935, *p* = 0.0065 on day 7) (Fig. 7e, f).

### The comparative organ distribution study using carbon black (CB) nanoparticles

**Fig. 8:**
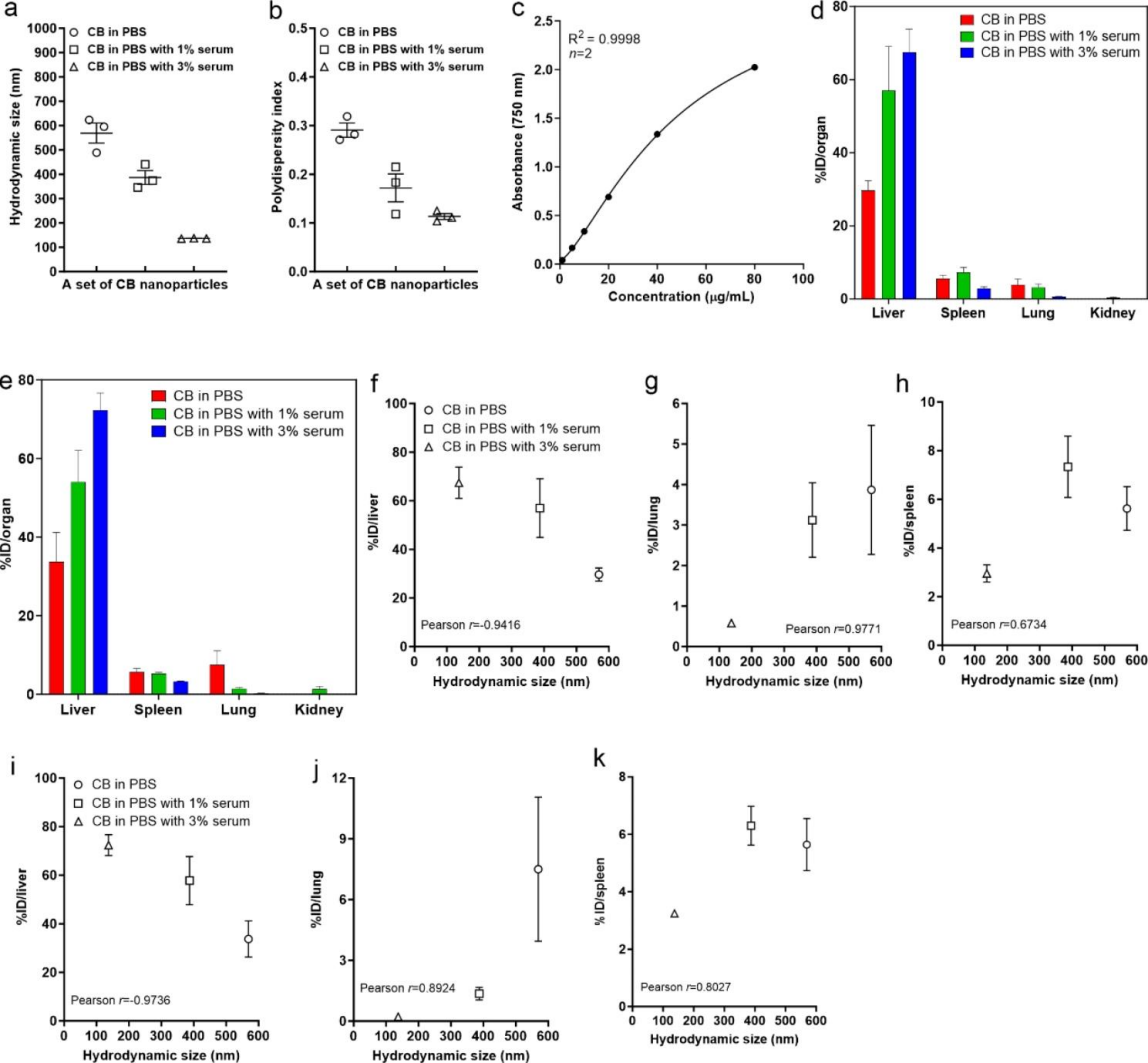
The organ distribution study of carbon black (CB) nanoparticles as a reference material. The hydrodynamic size of CB was controlled by dispersing them with different amounts of serum (i.e., 0, 1, and 3% mouse serum). The suspensions of CB with different hydrodynamic sizes were injected at 500 µg/mouse to eight-week-old female ICR mice via the tail vein. Then, the concentration of CB in organs was measured at days 1 and 7 by the identical method described for the ND. (**a**) The dispersion of CB with 0, 1, and 3% mouse serum produced a different set of hydrodynamic sizes. (**b**) The smaller hydrodynamic size showed more homogeneous dispersion. (**c**) The standard curve fit of CB samples by absorbance at 750 nm wavelength. The organ distribution of CB at (**d**) 1 and (**e**) 7 days post-injection. The Pearson correlation test between the hydrodynamic size and the percentage of initial treatment dose (%ID) per organ at day 1 [(**f**) liver, (**g**) lung, and (**h**) spleen] and 7 [(**i**) liver, (**j**) lung, and (**k**) spleen]. The correlation patterns between the hydrodynamic size and concentration of particles in organs were similar to those of NDs. Data are expressed as mean ± SEM for each group (*n* = 3 per group for hydrodynamic size and polydispersity; *n* = 2 for the standard curve fit; *n* = 4 per group for organ distribution of CB)

The CB nanoparticles were used as a reference material to perform a comparative organ distribution study by controlling hydrodynamic size. The controlled hydrodynamic sizes by dispersing them with different amounts of serum (i.e., 0, 1, and 3% mouse serum) were 569.4, 386.9, and 136.5 nm, respectively (Fig. 8a). The smaller hydrodynamic size showed better polydispersity, which implies smaller particles has better dispersion (Fig. 8b). The quantification of CB in dimethyl sulfoxide (DMSO) using the UV-Vis spectrophotometer showed an excellent standard curve fit with high sensitivity (Fig. 8c). The concentrations of CB in organs at days 1 and 7 showed similar results with those of ND studies (Fig. 8d and e). The Pearson correlation study between the hydrodynamic size and concentration of particles in organs showed a similar pattern with excellent correlation coefficients (Fig. 8f – k). Therefore, the findings shown in the ND study may not be specific to ND but also can apply to CB nanoparticles.

## Discussion

NDs have emerged as promising candidates for drug delivery platforms and bioimaging probes in the biomedical field because of their unique physicochemical properties and excellent biocompatibility [3]. However, although there have been extensive investigations of NDs in biomedical applications, the tissue distribution kinetics of NDs are lacking because of various difficulties, including quantification and controlled synthesis. Therefore, as shown in this study, the distribution kinetics of NDs *via* intravenous injection can provide critical information on the efficient and safer-by-design of NDs in biomedical applications.

In this study, precise control of the *sp*^*3*^*/sp*^*2*^ carbon ratio without significant changes in the size, shape, and surface area was possible because the panel of NDs originated from the same [19]. Thus, the selection of these two types of NDs is a good model for evaluating the effect of the *sp*^*3*^*/sp*^*2*^ carbon ratio. UV-Raman characterization showed that the diamond peak at 1325 cm^− 1^ significantly increased and the G peak strongly upshifted from 1590 cm^− 1^ in ^high^ND. In addition, the color difference between ^low^ND and ^high^ND distinctively demonstrated that ^low^ND contains more graphite [22, 23]. Furthermore, the XRD pattern again confirms that the ^low^ND has more graphite because the broad peak at 2 θ = 26° corresponds to graphite [24]. Finally, the HR-TEM images show that ^low^ND has a higher number of graphitic shells covering the diamond cores than the ^high^ND.

The quantification of carbon nanomaterials in organs is difficult but possible using various methods, such as thermal-optical analysis and mass spectrometry [25, 26]. Furthermore, NDs among carbon nanomaterials are more challenging to measure concentrations distributed in organs. In this regard, an efficient and straightforward method for quantifying NDs in organ samples has been developed. Our previous study proposed a new approach to measure the organ burden of low *sp*^*3*^*/sp*^*2*^ NDs using PK and UV-Vis spectrophotometry [27]; however, this study further suggested that this method can be broadly applied to any ND type. Furthermore, the LOQ of NDs (10 µg/mL) in this study is sufficient for the organ burden of NDs, considering the high injection dosage for in vivo injection [27–29].

In this study, the intravenous injection of NDs exhibited a fast distribution to the liver, spleen, and lungs within 30 min post-injection and persisted for up to 28 days. Accumulation in organs did not differ according to the *sp*^*3*^*/sp*^*2*^ carbon ratio. Although the *sp*^*3*^*/sp*^*2*^ carbon ratio determines the inflammation potential of NDs because *sp*^*2*^ carbon is the main source of reactive oxygen species [19], the tissue distribution pattern is not related to oxidative stress but to hydrodynamic size. The three accumulating organs of NDs, the liver, spleen, and lungs, shown in this study, are unique and different from other nanomaterials in previous studies [30–33]. For example, intravenously injected gold and silica nanoparticles accumulated in RES organs; however, particles were eliminated *via* the biliary or urinary routes, which was not observed in this study [30, 34]. In addition, single-walled carbon nanotubes (SWCNT) after intravenous injection showed an accumulation propensity in the liver, spleen, and lung; however, there was a decreasing tendency in time-course accumulation in all three organs, which is inconsistent with our findings [35].

Furthermore, it was noted that the injected NDs were hardly excreted, as the combined levels in the liver, spleen, and lungs were almost consistent throughout the study period (i.e., 28 days). A previous study with ND also suggested that ND has accumulating propensity in the liver and lung over 28 days without significant excretion via urine or feces [36]. The extreme accumulation pattern of NDs without any adverse effects can be advantageous for biomedical applications, particularly in theragnostic applications, because they can be used for surveillance and therapy at any time [37, 38]. For example, the controlled deposition and persistence of NDs without any histological alterations in the RES organ can provide excellent properties for cancer therapy from primary to metastatic tumors [39, 40]. However, further investigations on the theragnostic approach of NDs are needed.

In this study, agglomerated NDs were deposited in the alveolar capillaries of the lungs, which is consistent with previous studies that used metal oxides, gold, silver sulfide, and SWCNT [41–44]. However, this study suggests that the hydrodynamic size can control the lung deposition pattern. The pulmonary distribution of NDs is not in the alveoli or interstitium but in the alveolar capillaries. In this study, all four types of ND samples have the same primary particle size; the only difference is the percentage of serum to disperse particles. Because all ND samples interact immediately with the serum protein when injected intravenously, even bare-conditioned ND particles bind with various proteins, leading to a protein corona formation [45]. Therefore, NDs dispersed in PBS will be encapsulated by a protein corona, which means all NDs used in this study have a serum-coated interface in vivo, and the only difference between ND and SND is agglomeration size. Furthermore, the deposited NDs in the lung were re-distributed to secondary organs such as the spleen and liver. Based on current knowledge, most particles accumulate rapidly in the liver and spleen as the particle size increases [46]. In contrast, most particles accumulate more in the kidney as the particle size decreases [12, 47–49]. The size of particles that determines organ distribution and clearance depends on whether they can penetrate biological barriers, such as capillaries and endothelium. Specifically, Blanco et al. suggested that particles > 150 nm are more likely to be entrapped in the liver and spleen [47]. Danaei et al. suggested that nanocarriers with 100–150 nm diameter are distributed in the kidney and lung, whereas nanocarriers with 20–100 nm diameter may be distributed to the spleen, liver, and some secondary organs with leaky capillaries [48].

Nanoparticles injected intravenously accumulate less in the liver and distribute wider to multiple organs when the particle size is smaller. However, most of these findings are proved based on the primary size rather than hydrodynamic size [12]. On the other hand, there are contradictory findings, which suggest that smaller particles do not always show less accumulation in the liver, and the extent of liver accumulation can vary regardless of size in some cases. For example, the intravenous administration of Al_2_O_3_ showed a decreasing accumulation level in the liver as increasing particle size from 40 nm to 10,000 nm [43]. Likewise, a kinetics study of various sizes of polystyrene nanoparticles (i.e., 25, 50, 100, 200, and 500 nm) showed that the 25 nm-sized particles have the lowest liver accumulation, but 50 nm-sized particles show the highest liver accumulation [50]. In addition, a kinetics study using various sizes of gold nanoparticles (i.e., 10, 50, 100, and 250 nm) showed that the highest and lowest accumulating particles in the liver were 10 nm- and 50 nm-sized particles, respectively [18]. These findings highlight that the primary particle size may not be a dominant factor determining liver accumulation for some types of particles. Unfortunately, there are few studies about the effect of the hydrodynamic size of nanoparticles on organ distribution via intravenous injection. However, a previous study using iron oxide nanoparticles by controlling hydrodynamic size with PEG coating showed that the smaller hydrodynamic size has a higher liver accumulation [51]. In addition, the organ distribution study using CB nanoparticles shown in this study as a reference material further highlights that the findings of the ND study may not be specific to NDs but also can apply to other nanoparticles such as CB nanoparticles. Therefore, the tissue distribution pattern of NDs by the hydrodynamic size shown in this study is a novel finding contributing to the biomedical application of nanomaterials.

Information regarding the size ranges determining organ distribution would be helpful in understanding the size effect on biodistribution over a broad scope. However, because the biological consequences can vary depending on factors such as the pore size of the fenestrated capillaries and the behavior of nanoparticles at the cellular level, the suggestion of size ranges for each nanoparticle is required to fulfill its function in the field of drug delivery systems. In this way, our findings of the threshold size limit of approximately 300 nm to evade pulmonary deposition can provide information on modulating the biokinetics of NDs.

## Conclusions

**Fig. 9:**
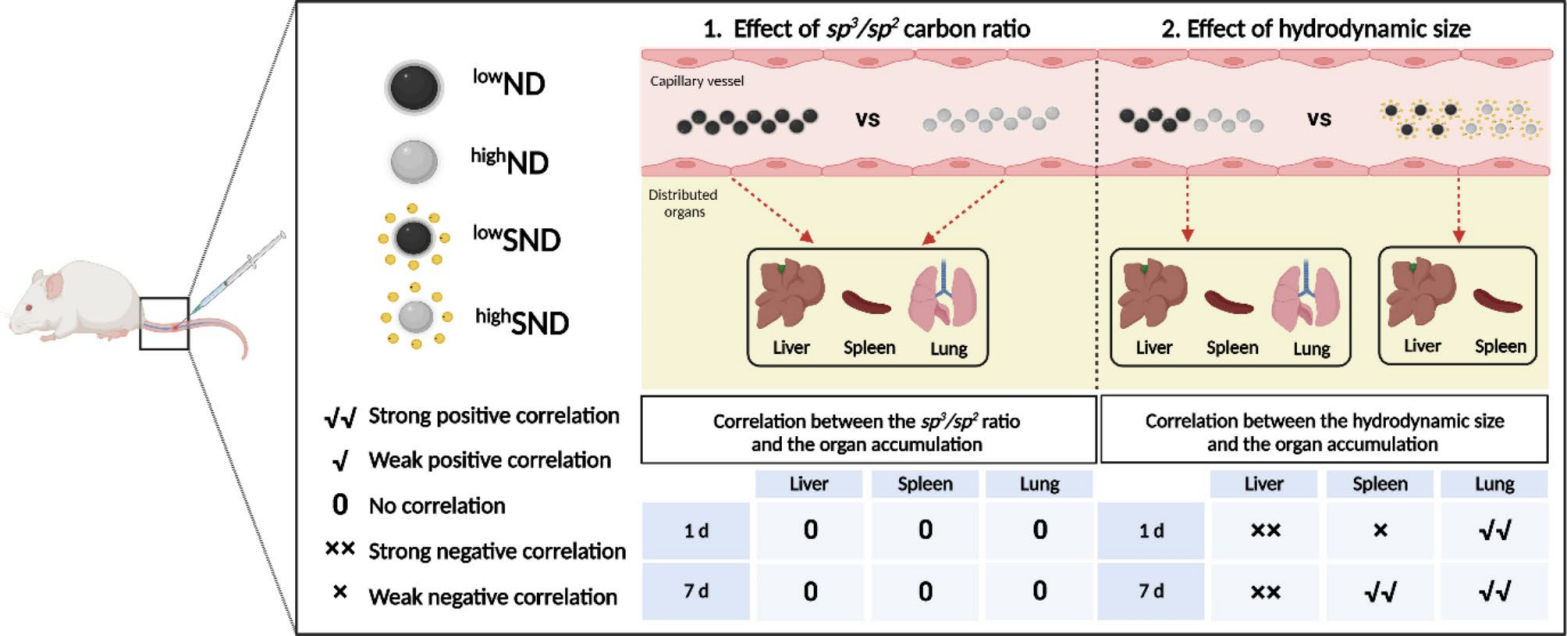
Diagram of the effects of *sp*^*3*^*/sp*^*2*^ carbon ratio and hydrodynamic size on the biokinetics of NDs injected into the tail vein of mice. No correlation of *sp*^*3*^*/sp*^*2*^ carbon ratio with the organ distribution levels was observed, suggesting that *sp*^*3*^ and *sp*^*2*^ carbon has no difference in the biokinetics of carbon particles. However, the hydrodynamic size showed excellent correlations with the major accumulating organs such as liver, spleen, and lungs, which suggests that hydrodynamic size is one of the major factors deciding the biokinetics of NDs

The intravenous injection of NDs into mice suggested new findings, including (1) *sp*^*3*^*/sp*^*2*^ carbon ratio is not a determinant of its tissue distribution, (2) combined levels of NDs in the liver, spleen, and lungs were approximately 100% of the ID for up to 28 days, (3) NDs are hardly excretable from the body for up to 28 days, (4) the hydrodynamic size of NDs, which is approximately 300 nm, can evade the pulmonary deposition (Fig. 9). Therefore, the distribution kinetics of NDs shown in this study can provide important information for biomedical applications of nanomaterials *via* intravenous injection.

## Materials and methods

### Preparation of NDs with low and high *sp*^*3*^*/sp*^*2*^ carbon ratio

In this study, we used detonation-synthesized NDs. To evaluate the effect of the *sp*^*3*^*/sp*^*2*^ carbon ratio on the biokinetics of NDs, low *sp*^*3*^*/sp*^*2*^ NDs (^low^ND) and high *sp*^*3*^*/sp*^*2*^ NDs (^high^ND) were prepared according to our previous study [52]. Briefly, 100 mL of HClO_4_ (70–72%, Duksan, Ansan, Korea) was added to a three-neck round-bottom flask. Thereafter, 1 g of ND soot (SW Chemicals Co., Ltd., Korea) was added, and the solution was stirred for at least 3 h. Thereafter, ^low^ND was prepared by incubating for 3 h at 135 °C, whereas ^high^ND was incubated for 24 h at 210 °C. After the reaction, the solution was cooled to room temperature and washed by centrifugation at 21,130 ×*g* for 20 min. A total of 5 rounds of washing steps were performed, and the final product was dried in a vacuum oven at 60 °C overnight.

### Physicochemical characterization of NDs

The physicochemical properties of the NDs were evaluated, including shape, primary size, surface area, *sp*^*3*^*/sp*^*2*^ carbon ratio, surface charge, and hydrodynamic size. HR-TEM (Tecnai G2 F20 460 L, Hillsboro, USA) was used to evaluate the shape and size of NDs. The specific surface areas of the NDs were estimated using the Brunauer-Emmett-Teller (BET) method (BELSORP-max, BEL Japan). The *sp*^*3*^*/sp*^*2*^ carbon ratio was measured using a Renishaw confocal Raman spectrometer (Renishaw, Gloucestershire, UK). XRD spectra were obtained to evaluate the graphite and diamond peaks for ND samples. XRD was performed using a PANalytical Empyrean (Almelo, Netherlands) with Cu-Kα radiation (λ = 0.1540598 nm) at a scanning rate of 0.026° per step. In addition, the surface charge, hydrodynamic size, and polydispersity of the NDs under various dispersion conditions were measured using a Zetasizer Nano-ZS (Malvern, Malvern Hills, UK).

### The surface charge and hydrodynamic size measurement of ND samples before and after incubation with mouse plasma

The zeta potential and hydrodynamic size of ND samples before and after incubation with mouse plasma were measured to evaluate how the as-prepared particles changed their size and zeta potential after intravenous injection into mice. Briefly, all as-prepared ND samples for intravenous injection were incubated with mouse plasma at 37 °C water bath for 30 min, and the particles were collected by centrifugation for 1 h at 21,130 ×*g* and redispersed in DW. The plasma was prepared by collecting the whole blood of 8-week-old female ICR mice (Samtako, Gyeonggi-do, Korea) with sodium citrate (Sigma-Aldrich, St. Louis, MO, USA). ND samples (100 µL) at 2.5 mg/mL were incubated with 100 µL of plasma prepared by centrifugation at 376 ×g for 5 min. The concentration of 2.5 mg/mL for incubation with mouse plasma was the same as the working concentration for mouse intravenous injection. Then, the redispersing particles were completed by the sonication for 10 min in a bath sonicator (Saehan-Sonic), and the zeta potential and hydrodynamic size were measured using a Zetasizer Nano-ZS (Malvern).

### Preparation of ND samples for in vivo studies

Pristine ^low^ND and ^high^ND samples and ^low^SND and ^high^SND were injected into the tail vein of mice to evaluate the effect of the *sp*^*3*^*/sp*^*2*^ carbon ratio and hydrodynamic size on the organ distribution kinetics of the NDs. The ND samples for intravenous injection were prepared using a modified method from our previous study [27]. Briefly, NDs dispersed in PBS for ^low^ND and ^high^ND were prepared by dispersing NDs at a test concentration and were sonicated for 30 min using a bath sonicator (Saehan-Sonic, South Korea). Meanwhile, NDs dispersed in PBS with 3% serum for ^low^SND and ^high^SND were prepared by dispersing in distilled water at a 5-fold higher concentration of working concentration and sonicated for 10 min in a bath sonicator. Thereafter, heat-inactivated mouse serum collected from the healthy female ICR mice was added at 3% v/v for working concentration and sonicated for 10 min. Finally, PBS corresponding to the working concentration was added and sonicated for 10 min.

### Animals and husbandry and intravenous injection of NDs into mice

Eight-week-old female ICR mice were purchased from Samtako (Gyeonggi-do, Korea) and housed in an individually ventilated cage system with controlled temperature (22 ± 1 °C), humidity (50 ± 10%), and a 12 h light/dark cycle. Food and water were provided *ad libitum*. All animals were acclimatized for seven days before the start of the experiment, and the animal experiments were approved by the Institutional Animal Care and Use Committee at Dong-A University (IACUC-DAU21-1). Although the dispersion stability of NDs was maintained for 2 h (Fig. S6, see Supporting Information), the particle samples were injected immediately after preparation. To select a dose for biokinetics, ND samples were injected at 100, 500, and 1,000 µg/mouse, and no significant changes in clinical signs, gross lesions, or histopathology was noted. Therefore, the injection dose was 500 µg/mouse. Furthermore, the time points were selected for up to 28 days because there were no histopathological changes in organs such as the liver, spleen, and lungs (Fig. S4, see Supporting Information). Therefore, four time points ( 30 min, 1 day, 7 days, and 28 days) were selected to evaluate the organ distribution kinetics of the NDs after a single intravenous injection.

### Collection of NDs in organs

At designated time points, the mice were sacrificed using isoflurane anesthesia (Piramal Critical Care, Bethlehem, PA, USA) under a rodent anesthesia system (VetEquip, Pleasanton, CA, USA). The collected organs were the liver, spleen, lungs, kidneys, heart, mesenteric lymph nodes, and brain. The collection of NDs from organs was performed according to a previously described method [27]. Briefly, the dissected organs were cut into small pieces (e.g., 1 cm ×1 cm cubic) and dried in an oven at 56℃ for 2 days. Thereafter, tissue digestion was performed by incubating 1 mL of PK (200 µg/mL; Promega, Madison, WI, USA) to 0.02 g of dried tissues in a tissue digestion buffer (30 mM Tris-HCl, 10 mM EDTA, 1% SDS, 5 mM CaCl_2_, pH8.0). The first round of tissue digestion was performed at 56℃ for 24 h, and particles and tissue remnants were collected by centrifugation for 1 h at 21,130 ×*g*. Subsequently, the second round of tissue digestion was performed by dispersing pellets with the same PK and incubation conditions. The organs, excluding the spleen, showed excellent digestion with two rounds of tissue digestion but tissue remnants were found in the spleen. Thus, one more tissue digestion was performed for the spleen with an alkaline tissue solubilizer (Solvable®; PerkinElmer, Waltham, MA, USA) by dispersing particles and tissue pellets with 0.5 mL of Solvable® and incubating at 50℃ for 24 h.

### Quantification of collected NDs

After the final round of tissue digestion, the samples were centrifuged at 21,130 ×*g* for 1 h to collect the particle pellets. Thereafter, pellets were dispersed in DMSO and sonicated for 30 min using a bath sonicator (Saehan Sonic). The collected NDs were quantified according to a previously described method [27]. NDs were quantified using a spectrophotometer (Lambda 365; Perkin Elmer, Waltham, MA, USA) because the absorbance of NDs at 750 nm can quantify carbon-based nanoparticles with minimal interference from biological components [27, 53]. The particle concentration was calculated using the standard curve fit of the NDs serially diluted in DMSO (Fig. S2, see Supporting Information). The absorbance value of the tissue lysates of ND-treated mice was subtracted from that of the vehicle control group to obtain the absorbance value of the particles without tissue remnants.

### Histopathology and dark-field microscopy

The mice were perfused with PBS to exclude the interference of erythrocytes in dark-field microscopy. Thereafter, organs were fixed in 10% neutral buffered formalin and processed for the routine histological slides with equipment at the Neuroscience Translational Research Solution Center (Busan, South Korea). The collected organs were the liver, spleen, lungs, kidneys, mesenteric lymph nodes, brain, and heart. The slides were stained with hematoxylin and eosin (H&E), and histopathological observations were performed. In addition, the slides were lightly stained with PSR, and the tissue distribution of NDs was evaluated using dark-field microscopy (Nikon, Tokyo, Japan).

### Observation of NDs distribution by TEM

Lungs were dissected into 1 mm^3^ cubes after perfusion with 4% paraformaldehyde in PBS and fixed with 2.5% glutaraldehyde in 0.1 M phosphate buffer. Sample preparation, including processing and microdissection, was performed at the Korea Basic Science Institute (KBSI; Cheongju, Korea). The localization of NDs in the lung tissue was evaluated using a Talos F200X Field Emission Scanning Transmission Electron Microscope (FE-STEM; Thermo Fisher Scientific; Waltham, MA, USA) equipped in the center for collaborative instruments at Dong-A University (Busan, Korea).

### Comparative study using CB nanoparticles

As a reference particle, CB nanoparticles were tested for the comparative organ distribution pattern. Briefly, a set of CB (Printex 90, Evonik Degussa GmbH, Frankfurt, Germany) with different hydrodynamic sizes was prepared by dispersing them with different amounts of serum (i.e., 0, 1, and 3% mouse serum). The suspensions of CB with controlled hydrodynamic sizes (i.e., 569.4, 386.9, and 136.5 nm, respectively) were injected at 500 µg/mouse to eight-week-old female ICR mice via the tail vein. Then, the concentration of CB in organs was measured at days 1 and 7 by the identical method described for the ND. The non-linear standard curve fit of CB dispersed in DMSO showed excellent goodness of fit (R^2^ = 0.9998) with high sensitivity (Fig. 8c).

### Statistical analysis

GraphPad Prism software (ver. 9.0; La Jolla, CA, USA) was used for graphs and statistical analysis. The data are expressed as mean ± standard error of the mean (SEM). A paired t-test was applied to assess the statistical significance of the surface charge and hydrodynamic size values before and after incubation with mouse plasma. The values of toxicokinetic parameters were determined using descriptive statistics analysis and the calculation of the AUC by the trapezoidal rule method. The correlation analysis between the hydrodynamic size and the percentage of the injected dose per organ was performed by applying a two-tailed Pearson correlation test.

### Electronic supplementary material

Below is the link to the electronic supplementary material.

Supplementary Material 1

## Data Availability

The original contributions presented in the study are included in the article. Further inquiries can be directed to the corresponding authors.

## List of Abbreviations

AUC: Area under the curve
CB: Carbon black
C_max_: Concentration max
ND: Nanodiamond
^low^ND: Nanodiamond with low *sp*^*3*^*/sp*^*2*^ carbon ratio
^high^ND: Nanodiamond with high *sp*^*3*^*/sp*^*2*^ carbon ratio
^low^SND: Serum-coated ^low^ND
^high^SND: Serum-coated ^high^ND
HR-TEM: High-resolution transmission electron microscopy
PBS: Phosphate-buffered saline
PEG: Polyethylene glycol
PSR: Picrosirius red
RES: Reticuloendothelial system
SAED: Selected-area electron diffraction
SEM: Standard error of the mean
SWCNT: Single-walled carbon nanotubes
T_max_: Time max
XRD: X-ray diffraction
