# Supplementary material for: Effect of sp3/sp2 carbon ratio and hydrodynamic size on the biodistribution kinetics of nanodiamonds in mice via intravenous injection
Source: Part Fibre Toxicol. 2023 Aug 21;20:33. doi: 10.1186/s12989-023-00545-7 (PMC10440929; doi:10.1186/s12989-023-00545-7)
Supplement: Supplementary file 1 — Supplementary Material 1 [file 12989_2023_545_MOESM1_ESM.docx]

Supplementary file

Effect of *sp^3^/sp^2^* carbon ratio and hydrodynamic size on the biodistribution kinetics of nanodiamonds in mice via intravenous injection

Jiyoung Jeong^1^, Soyeon Jeon^1^, Songyeon Kim^1^, Sinuk Lee^1^, Gyuli Kim^1^, Eunsol Bae^1^, Yeonjeong Ha^1^, Seung Whan Lee^2^, Ji-Su Kim^3^, Dong-Jae Kim^4^, and Wan-Seob Cho^1*^

^1^Lab of Toxicology, Department of Health Sciences, Dong-A University, Busan 49315, Republic of Korea

^2^Plasma Technology Research Center, National Fusion Research Institute, Gunsan-si 54004, Republic of Korea

^3^Primate Resources Center (PRC), Korea Research Institute of Bioscience and Biotechnology (KRIBB), Jeongeup, 56216, Republic of Korea

^4^Laboratory Animal Resource Center, DGIST, Daegu 42988, Republic of Korea

**^*^Corresponding author:**

Professor Wan-Seob Cho

Lab of Toxicology, Department of Health Sciences, The Graduate School of Dong-A University, 37, Nakdong-dero 550 beon-gil, Saha-gu, Busan 49315, Republic of Korea.

Tel: +82-51-200-7563, E-mail: [wcho@dau.ac.kr](mailto:wcho@dau.ac.kr)

Email addresses:

JJ: [dudwlwjd@naver.com](mailto:dudwlwjd@naver.com); SJ: [wjsthdus0418@naver.com](mailto:wjsthdus0418@naver.com); SK: [songyounkim@naver.com](mailto:songyounkim@naver.com); SL: [dodokook@naver.com](mailto:dodokook@naver.com); GK: [rbfl6692@naver.com](mailto:rbfl6692@naver.com); EB: [eunsol0731@naver.com](mailto:eunsol0731@naver.com); YH: [gkduswjd1101@naver.com](mailto:gkduswjd1101@naver.com); SWL: [leesw@nfri.re.kr](mailto:leesw@nfri.re.kr); JSK: [kimjs@kribb.re.kr](mailto:kimjs@kribb.re.kr); DJK: [kimdj@dgist.ac.kr](mailto:kimdj@dgist.ac.kr); WSC: [wcho@dau.ac.kr](mailto:wcho@dau.ac.kr)

**
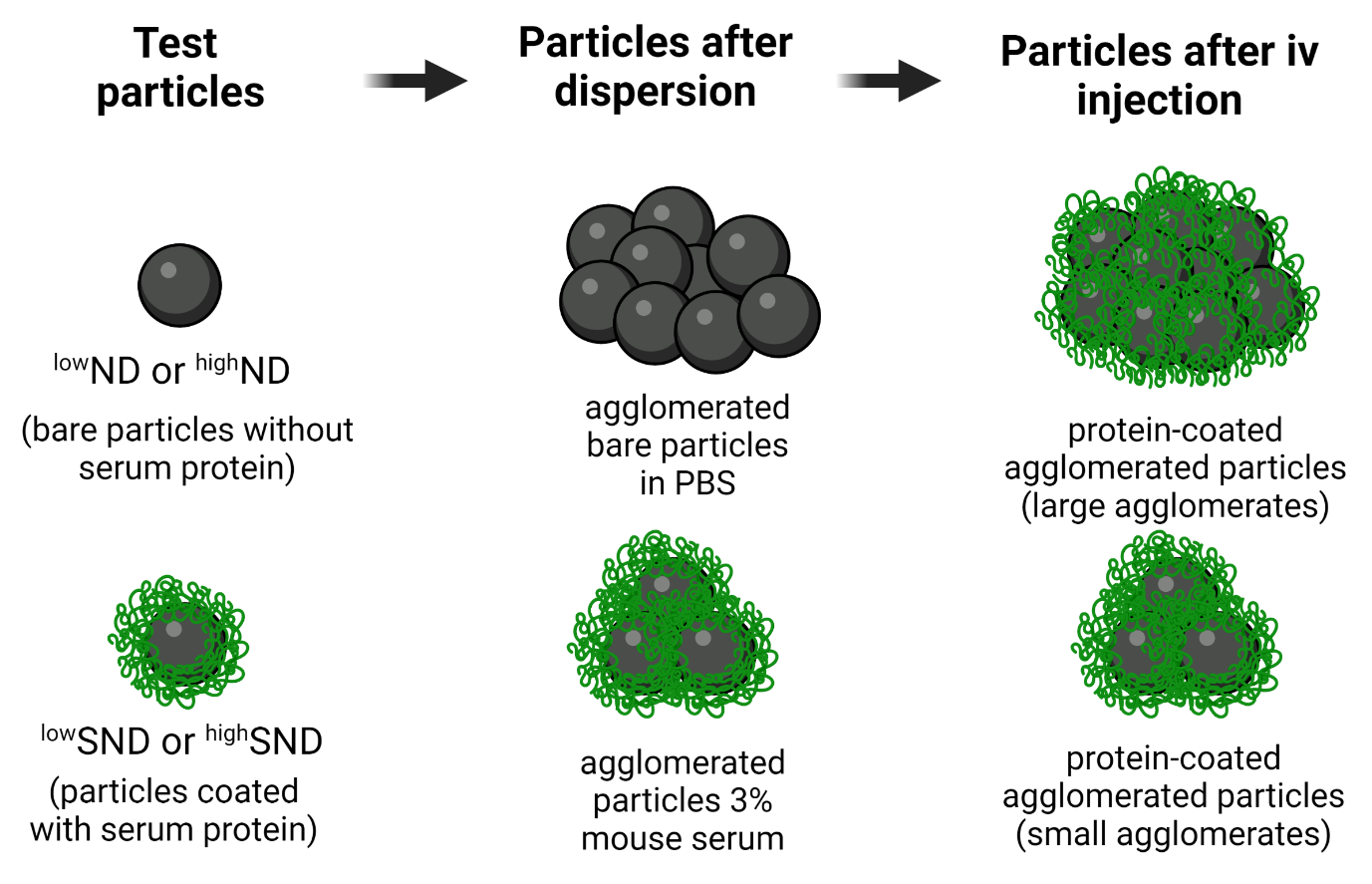
**

**Fig. S1.** The hypothetical illustration of serum protein coating on the test nanoparticles. In case the bare particles such as ^low^ND and ^high^ND are injected without protein coating, they will immediately adsorb protein on the surface of agglomerated particles. On the other hand, the protein-coated particles before injection will not severely adsorb protein on the surface of the particles. Thus, particles with or without protein coating before intravenous injection might have similar protein coating on their surface, but the agglomeration size will differ.


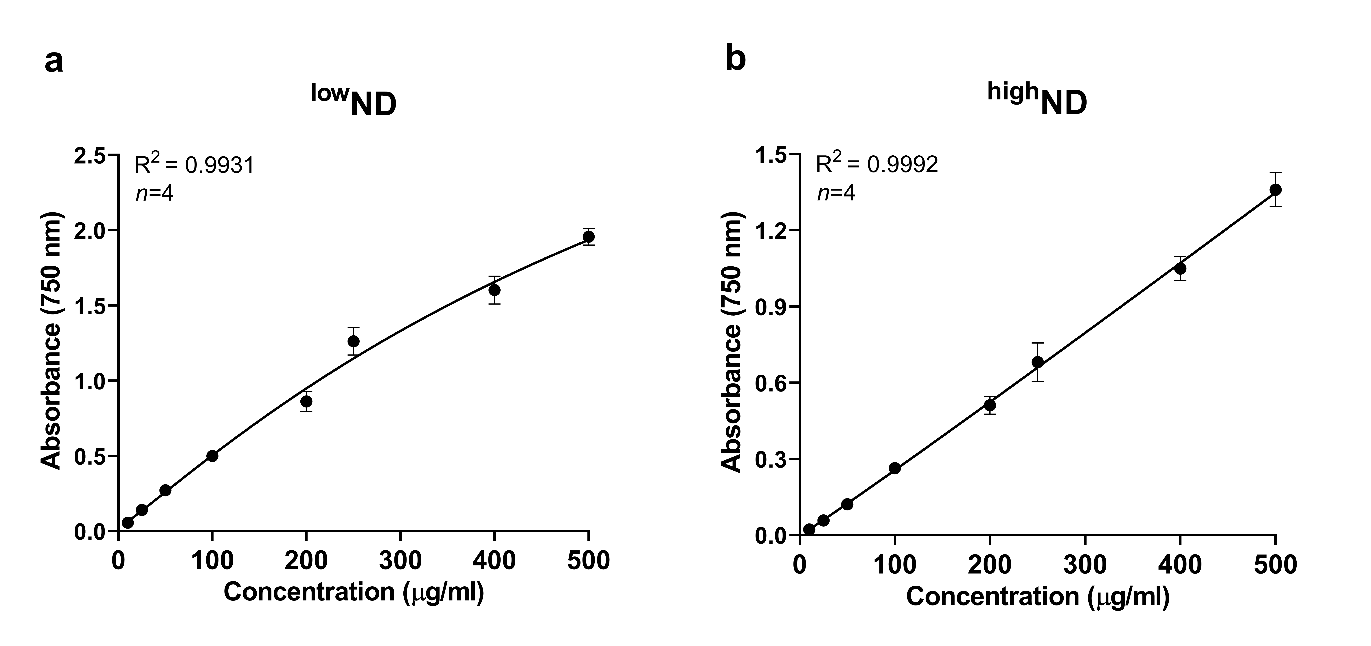
**Fig. S2.** The standard curve fit of (a) ^low^ND and (b) ^high^ND using a UV-Vis spectrophotometer. Note that each nanodiamond (^low^ND and ^high^ND) was dispersed in dimethyl sulfoxide (DMSO), and absorbance was measured at 750 nm. The data are expressed as mean ± SEM (*n*=4) for each group.


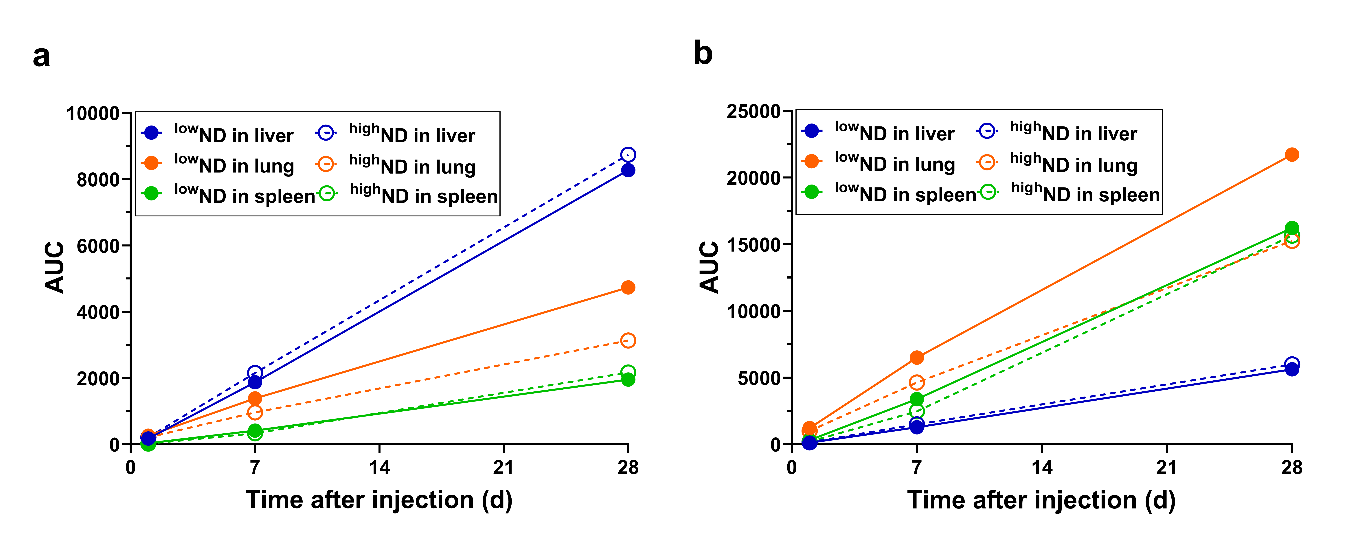
 **Fig. S3.** Time-dependent area under the curve (AUC) of the liver, spleen, and lungs. The AUC levels in these organs were calculated by (a) concentration per organ or (b) concentration per organ weight. Data are expressed as mean ± SEM (*n*=5) for each group.


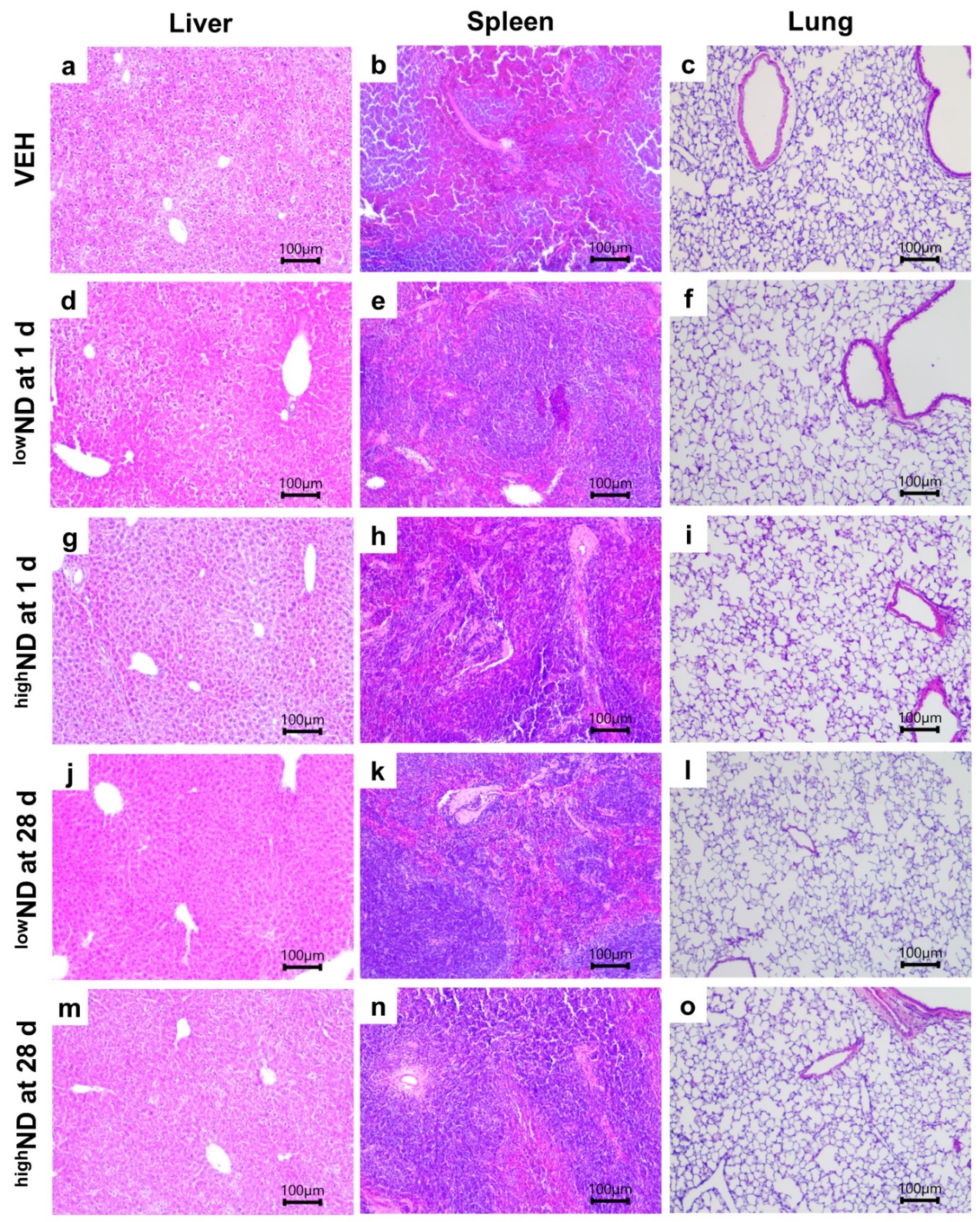
**Fig. S4.** Histopathological analysis in the liver, spleen, and lungs at day 1 and day 28 post-injection of nanodiamonds (^low^ND and ^high^ND) at 500 μg/mouse. Liver, spleen, and lung section of mice in the (a-c) VEH group, (d-f) ^low^ND group at day 1, (g-i) ^high^ND group at day 1, (j-l) ^low^ND group at day 28 and (m-o) ^high^ND group at day 28. Note that intravenous injection of nanodiamonds at both time points showed no histological changes comparable to the vehicle control group (VEH). Scale bar=100 μm.


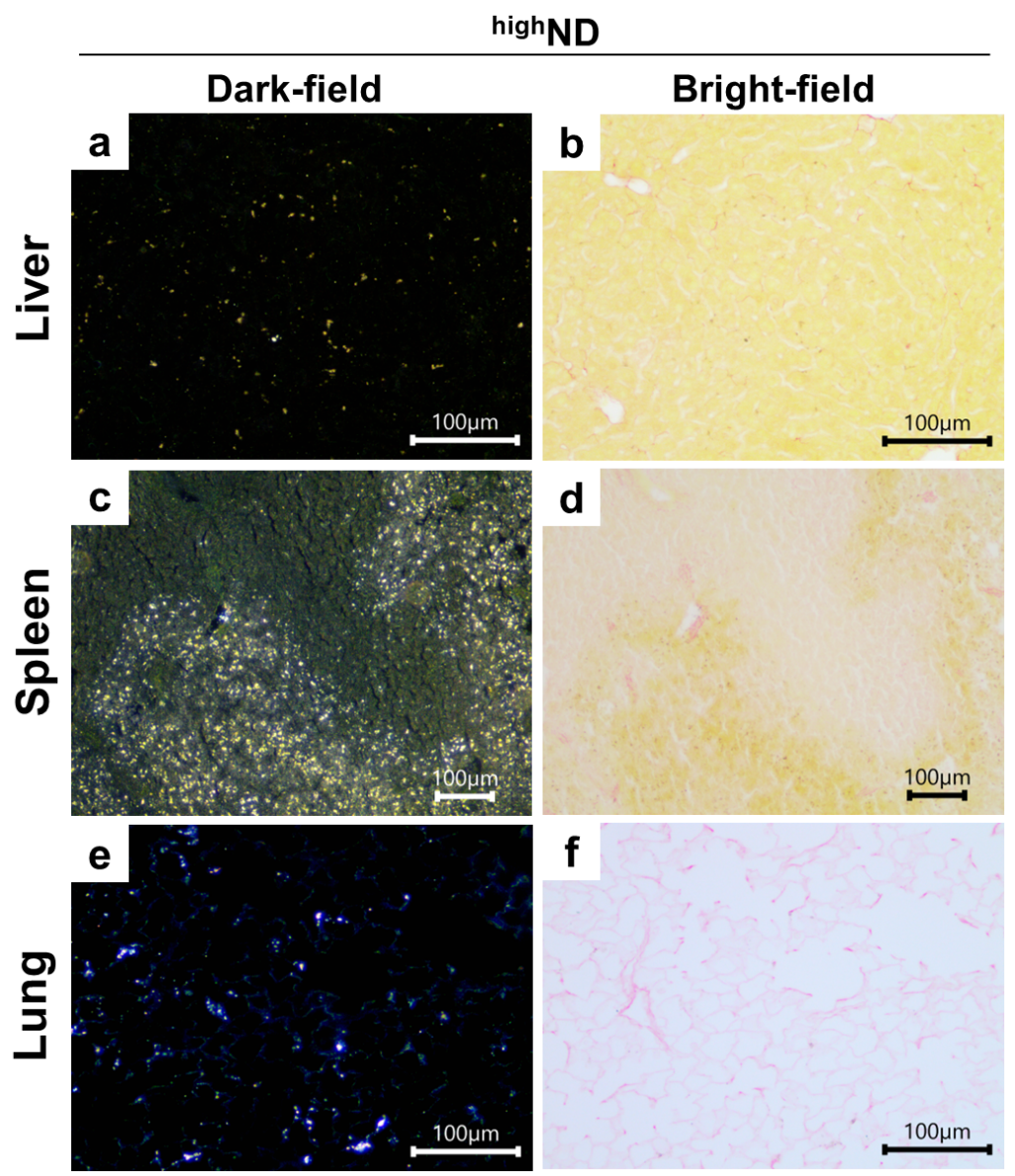
**Fig. S5.** Representative dark-field and bright-field images of the liver, spleen, and lung at day 7 post-injection of ^high^ND. Liver images of (a) dark- and (b) bright-field microscopy. Spleen images of (c) dark- and (d) bright-field microscopy. Lung images of (e) dark- and (f) bright-field microscopy. The dark-field and bright-field images were taken from the same slides. Scale bar=100 μm.


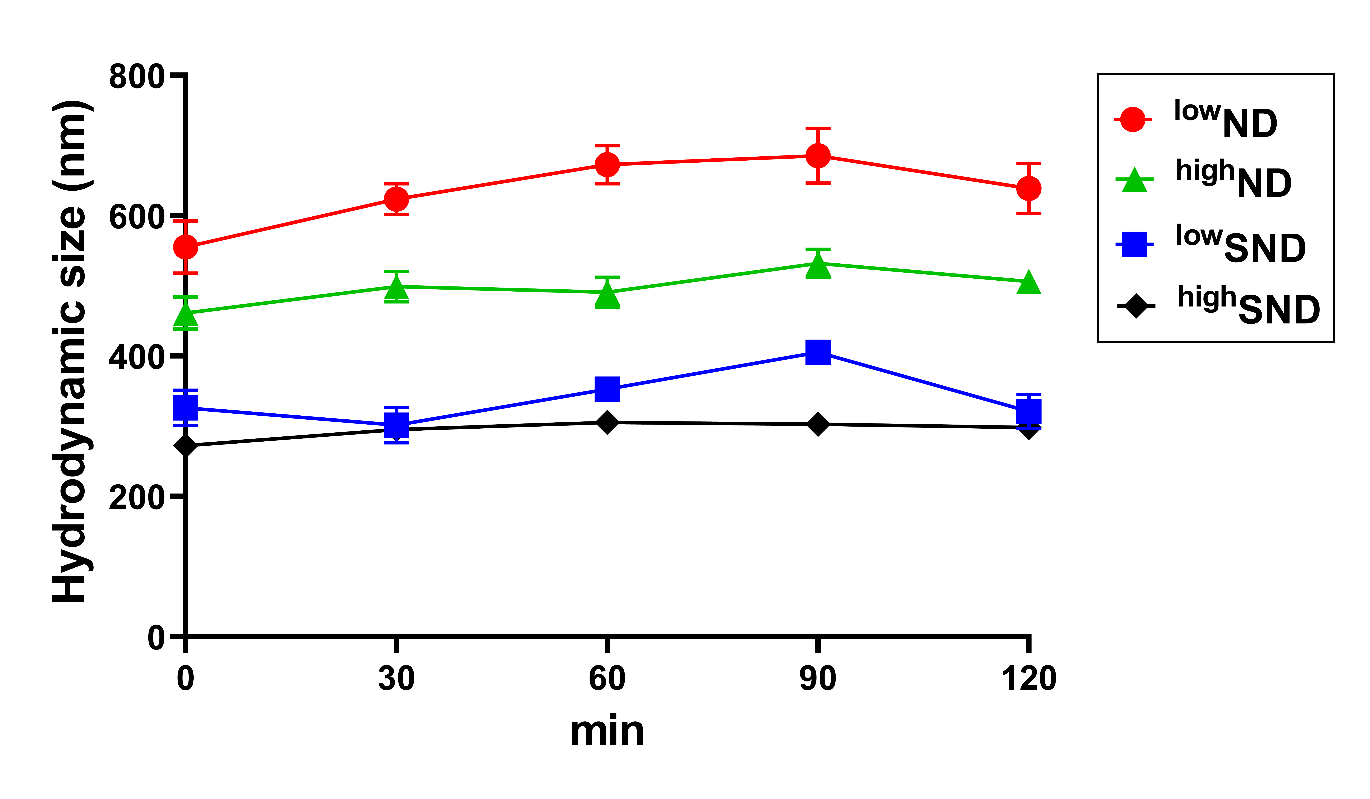


**Fig. S6.** The dispersion stability of nanodiamonds without serum coating (^low^ND and ^high^ND) and with serum coating (^low^SND and ^high^SND). The data are expressed as mean ± SEM (*n*=3) for each group.

**Table S1.** The concentration of nanodiamonds per organ

| Time after injection | 30 min | 1 d | 7 d | 28 d |
| --- | --- | --- | --- | --- |
| Concentrations of ^low^ND in organ | | | | |
| - Liver | 84.4 ± 10.6 | 266.0 ± 40.7 | 300.0 ± 50.3 | 309.0 ± 27.3 |
| - Spleen | 21.4 ± 8.3 | 46.0 ± 7.3 | 81.4 ± 21.7 | 65.1 ± 11.0 |
| - Lung | 296.0 ± 44.7 | 230.0 ± 52.9 | 145.0 ± 49.1 | 174.0 ± 72.9 |
| - Kidney | <LOQ | <LOQ | <LOQ | <LOQ |
| - MLN | <LOQ | <LOQ | <LOQ | <LOQ |
| - Brain | <LOQ | <LOQ | <LOQ | <LOQ |
| - Heart | <LOQ | <LOQ | <LOQ | <LOQ |
| Concentrations of ^high^ND in organ | | | | |
| - Liver | 104.0 ± 22.3 | 320.0 ± 47.7 | 326.0 ± 80.9 | 302.0 ± 96.4 |
| - Spleen | 14.0 ± 10.2 | 35.1 ± 8.1 | 67.9 ± 13.2 | 107.0 ± 29.7 |
| - Lung | 282.0 ± 69.3 | 141.0 ± 29.4 | 109.0 ± 16.1 | 97.6 ± 24.5 |
| - Kidney | <LOQ | <LOQ | <LOQ | <LOQ |
| - MLN | <LOQ | <LOQ | <LOQ | <LOQ |
| - Brain | <LOQ | <LOQ | <LOQ | <LOQ |
| - Heart | <LOQ | <LOQ | <LOQ | <LOQ |

The data were expressed as µg/organ (mean ± SEM). MLN; mesenteric lymph node, <LOQ; less than the limit of quantification (LOQ: 10 μg/mL for both particles).
